# Effects of mouthwash on periodontal pathogens and glycemic control in patients with type 2 diabetes mellitus

**DOI:** 10.1038/s41598-024-53213-x

**Published:** 2024-02-02

**Authors:** Saaya Matayoshi, Fumikazu Tojo, Yuto Suehiro, Makoto Okuda, Misato Takagi, Marin Ochiai, Maika Kadono, Yusuke Mikasa, Rena Okawa, Ryota Nomura, Yoshito Itoh, Naoto Itoh, Kazuhiko Nakano

**Affiliations:** 1 Joint Research Laboratory of Science for Oral and Systemic Connection, Osaka University Graduate School of Dentistry, 1-8 Yamada-Oka, Suita, Osaka 565-0871 Japan; 2 Department of Pediatric Dentistry, Osaka University Graduate School of Dentistry, Osaka, Japan; 3 Department of Pediatric Dentistry, Hiroshima University Graduate School of Biomedical and Health Sciences, Hiroshima, Japan; 4 Itoh Internal Medicine Clinic, Osaka, Japan; 5 Department of Endocrinology and Metabolism, Sumitomo Hospital, Osaka, Japan

**Keywords:** Microbiology, Medical research

## Abstract

Periodontitis is known to be associated with type 2 diabetes mellitus (T2DM), and gargling with mouthwash is known to reduce the incidence of periodontitis by inhibiting periodontal pathogens. However, the effects of mouthwash on oral and systemic conditions in patients with T2DM remain unknown. In this study, we investigated the effects of gargling with mouthwash on the number of red complex species, including *Porphyromonas gingivalis, Treponema denticola,* and *Tannerella forsythia*, and HbA1c levels in patients with T2DM. Patients were instructed to gargle with water for 6 months, followed by gargling with mouthwash containing chlorhexidine gluconate for the subsequent 6 months. At each clinic visit, saliva was collected and bacterial DNA was extracted to detect red complex species using the polymerase chain reaction technique. The HbA1c level was determined using a blood sample. The number of red complex species significantly decreased in younger or male patients who gargled with mouthwash. Furthermore, HbA1c levels significantly decreased in younger patients or patients with higher HbA1c levels who gargled with mouthwash. These results suggest that gargling with mouthwash reduces the number of red complex species and improves the hyperglycemic status in patients with T2DM, especially younger patients.

## Introduction

Periodontitis is a chronic inflammatory infectious disease caused by periodontopathic bacteria^1^. Clinical characteristics of periodontitis include the destruction of periodontal tissue and alveolar bone, which commonly leads to tooth loss in adults^2,3^. Periodontopathic bacterial species in the oral cavity are categorized according to their virulence. Red complex species including *Porphyromonas gingivalis, Treponema denticola,* and *Tannerella forsythia* are particularly virulent bacteria that play a major role in the progression of periodontitis^1,4,5^.

To inhibit periodontal pathogens, routine checkups and cleaning by dental professionals and daily self-care are important^6,7^. Of the various self-care products available, mouthwash is one of the easiest to use for those who want to improve their oral hygiene^8^. Mouthwash containing chlorhexidine gluconate is known to have antimicrobial effects on *P. gingivalis*^9^.

Periodontitis is also related to the development or progression of systemic diseases^10–13^. In fact, a bidirectional relationship between periodontitis and type 2 diabetes mellitus (T2DM) has been reported^14–18^. Patients with T2DM are more susceptible to severe periodontitis than subjects without diabetes, and inflammatory periodontitis aggravates hyperglycemia, leading to inadequate glycemic control^19^. Periodontitis and diabetes mellitus both affect many people worldwide. As the number of patients increases, the cost of medical care increases proportionally, and the number of patients who suffer social consequences, such as work restrictions due to treatment, also increases. Therefore, simple and innovative treatments are needed to reduce the number of people affected by both diseases^20,21^.

Recently, it has been reported that patients with T2DM treated for periodontitis have reduced periodontopathic bacteria and improved glycemic control^22–25^. Patients with T2DM complicated by periodontitis have more red complex species, and poor glycemic control is thought to be associated with increased levels of red complex species in the oral cavity^26^. However, the effects of mouthwash on oral and systemic conditions in patients with T2DM remain unknown.

In this study, we investigated the effects of gargling with mouthwash on the numbers of red complex species, including *P. gingivalis, T. denticola,* and *T. forsythia*, and HbA1c levels in patients with T2DM.

## Results

### Study design

**Figure 1:**
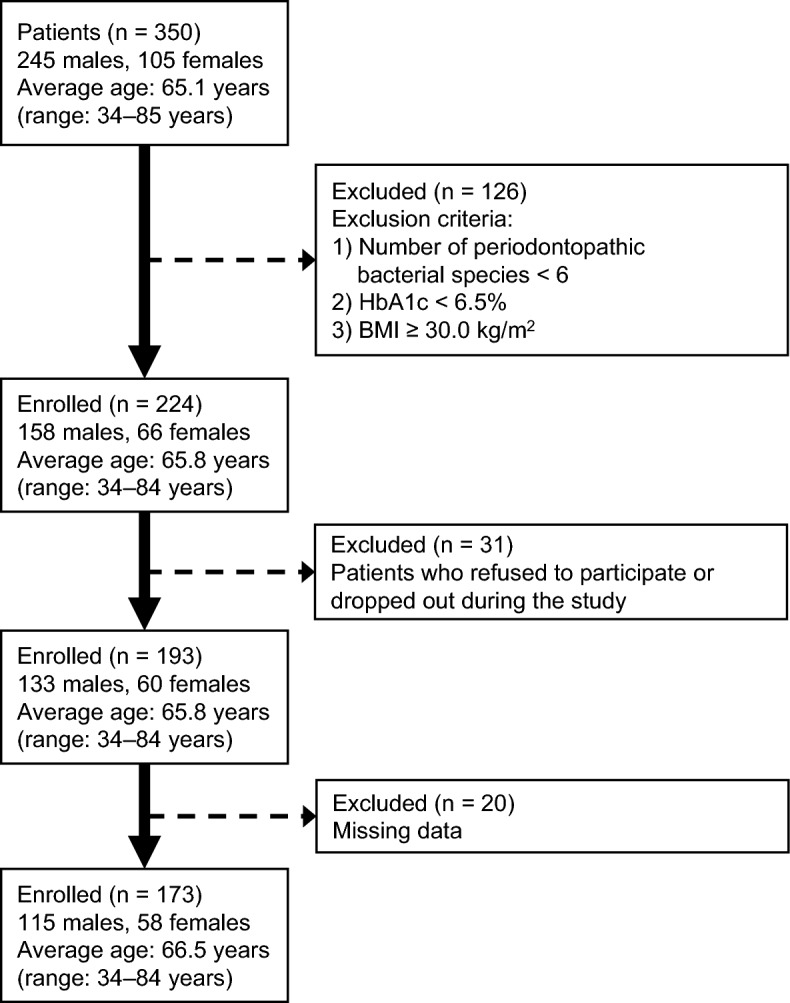
Flowchart of patient selection. *BMI* body mass index.

The flowchart of patient selection is shown in Fig. 1. This study started with 350 patients diagnosed with T2DM, and 224 were recruited by excluding those who met the exclusion criteria (total number of periodontal bacteria < 6, HbA1c < 6.5%, BMI ≥ 30.0 kg/m^2^) (Fig. 1). Subsequently, we excluded patients who dropped out and patients with missing data, resulting in a cohort of 173 patients. The characteristics of the 173 patients are shown in Table 1.

**Table 1:** Characteristics of the patients.

	Patients (n = 173)
Sex	
Male	115 (66)
Female	48 (34)
Age (years)	66.9 ± 0.79 [68]
HbA1c levels (%)	7.36 ± 0.07 [7.4]
Duration of T2DM (years)	13.46 ± 0.64 [13]
BMI (kg/m^2^)	23.66 ± 0.23

Values are n (%) or mean ± standard error of the mean [median].

**Figure 2:**
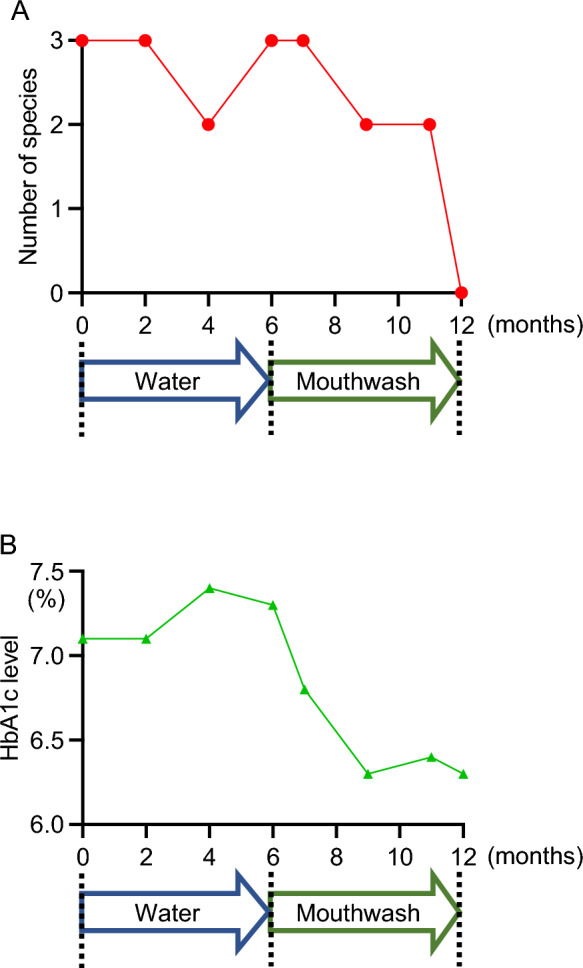
The time course of a remarkably effective case in this study. Chronological changes in the number of major periodontopathic bacterial species and red complex species (**A**) and HbA1c level (**B**). Time points for analysis were the start of water gargling, the end of water gargling, the start of mouthwash gargling, and the end of mouthwash gargling.

The study design is shown in Fig. 2. Patients were instructed to gargle with water three times a day for 6 months, followed by gargling three times a day with mouthwash for the next 6 months. During this experimental period, saliva specimens were collected 6–12 times, every 1–2 months at each visit to the clinic. Bacterial DNA was extracted from the saliva samples to detect the three red complex species (Fig. 2A). Additionally, HbA1c levels were determined by blood samples during the visit (Fig. 2B). In a remarkably effective case shown in Fig. 2, all red complex species were detected at the beginning of gargling with water and were not decreased at the end of gargling with water. After this time, the number of red complex species decreased during the 6 months of gargling with mouthwash and none were detected at the end of gargling with mouthwash. The HbA1c level increased slightly after gargling with water for 6 months, but decreased markedly after gargling with mouthwash for 6 months.

### Comparison of the number of red complex species as a function of gargling frequency

**Figure 3:**
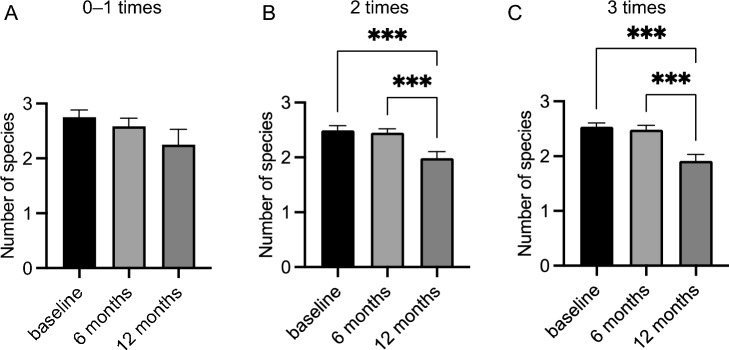
Comparison of the number of red complex species as a function of gargling frequency. Significant differences were observed using analysis of variance with Bonferroni correction (***P < 0.001).

Patients who gargled once a day or less (n = 12) showed no significant reduction in the number of red complex species after either water gargling or mouthwash gargling (Fig. 3A, Table 2). Patients who gargled twice a day (n = 80) or three times a day (n = 81) showed no decrease in the number of red complexes when gargling with water, but gargling with mouthwash significantly decreased the number of red complex bacteria (*P* < 0.001) (Fig. 3B and C, Table 2).

**Table 2:** Number of red complex species as a function of gargling frequency.

	Gargling frequency		
	Zero to one time (n = 12)	Two times (n = 80)	Three times (n = 81)
Baseline	2.75 ± 0.13	2.49 ± 0.08	2.54 ± 0.07
After 6 months	2.58 ± 0.14	2.45 ± 0.07***	2.48 ± 0.08***
After 12 months	2.25 ± 0.27	1.99 ± 0.12***	1.91 ± 0.12***

Values are mean ± standard error of the mean.

***P < 0.001 compared with baseline.

### Comparison of the number of red complex species and HbA1c levels

**Figure 4:**
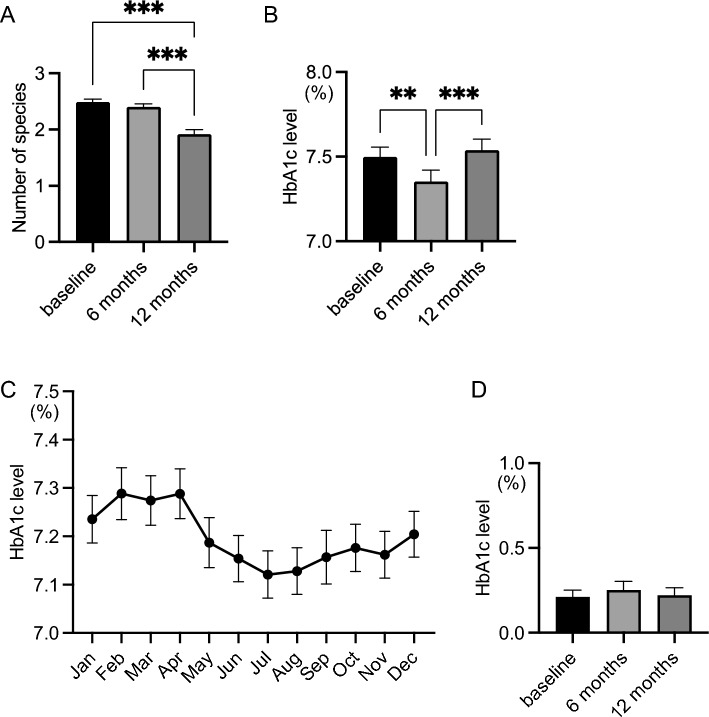
Comparison of the number of red complex species and HbA1c levels. (**A**) Number of red complex species. (**B**) HbA1c levels. (**C**) Seasonal variation of HbA1c levels. (**D**) HbA1c levels after excluding seasonal variation. Significant differences were observed using analysis of variance with Bonferroni correction (**P < 0.01, ***P < 0.001).

Because the number of red complex species did not decrease in patients who gargled once a day or less, we focused on the 161 patients who gargled two or three times a day. Gargling with mouthwash significantly decreased the number of red complex species (P < 0.0001), while gargling with water did not (Fig. 4A, Table 3). HbA1c levels decreased after gargling with water and increased after gargling with mouthwash (Fig. 4B, Table 3). HbA1c levels fluctuate seasonally^27,28^, and gargling with water and with mouthwash took place in different seasons. We checked the past HbA1c data in 161 patients and collected 9200 points-data in total which ranged from 0 to 8 (average 4.76) years (Fig. 4C, Table 4), which confirmed that gargling with water corresponded with the seasonal improvement period and gargling with mouthwash corresponded with the exacerbation period. To exclude this seasonal variation, we subtracted the average of past HbA1c values of the same months as measured in the study for each patient and analyzed the relationship with other factors from that point onwards. After this conversion, neither water gargling nor mouthwash gargling led to a significant reduction in HbA1c levels (Fig. 4D, Table 5).

**Table 3:** Number of red complex species and HbA1c levels.

	Number of red complex species	HbA1c levels (%)
Baseline	2.49 ± 0.05	7.50 ± 0.06
After 6 months	2.40 ± 0.05***	7.35 ± 0.07***
After 12 months	1.92 ± 0.08***	7.54 ± 0.07^†††^

Values are mean ± standard error of the mean.

***P < 0.001 compared with baseline.

^†††^*P* < 0.001 compared with after 6 months.

**Table 4:** Seasonal variation in HbA1c levels.

	HbA1c levels (%)
January	7.23 ± 0.05
February	7.29 ± 0.05
March	7.27 ± 0.05
April	7.29 ± 0.05
May	7.19 ± 0.05
June	7.15 ± 0.05
July	7.12 ± 0.05
August	7.13 ± 0.05
September	7.16 ± 0.06
October	7.18 ± 0.05
November	7.16 ± 0.05
December	7.20 ± 0.05

Values are mean ± standard error of the mean.

**Table 5:** Adjusted HbA1c levels.

	Adjusted HbA1c levels (%)
Baseline	0.21 ± 0.04
After 6 months	0.25 ± 0.05
After 12 months	0.22 ± 0.04

Values are mean ± standard error of the mean.

### Changes in the number of red complex species as a function of clinical factors

**Figure 5:**
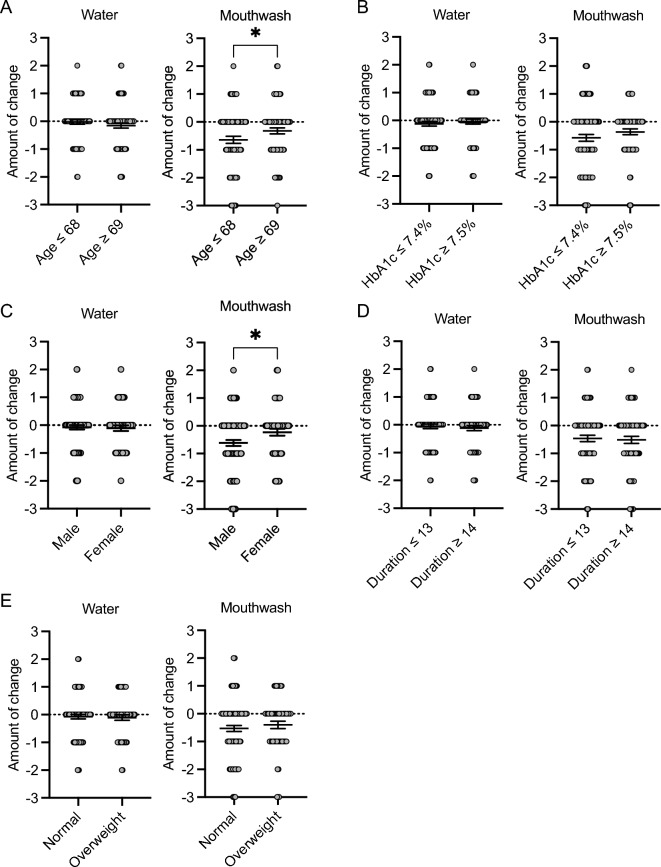
Comparison of changes in the number of red complex species as a function of clinical factors. (**A**) Age, (**B**) baseline HbA1c level, (**C**) sex, (**D**) disease duration, and (**E**) baseline BMI. Significant differences were observed using Student’s t-test (*P < 0.05).

To investigate the factors contributing to changes in the number of red complex species after gargling with water or mouthwash, we classified patients based on five clinical factors: age, baseline HbA1c levels, sex, disease duration, and baseline body mass index (BMI). After dividing the patients into two groups based on the median age (≤ 68 years, n = 83; ≥ 69 years, n = 78), there was no significant difference in the change in the number of red complex species between the two groups after gargling with water (Fig. 5A, Table 6). However, the number of red complex species was significantly lower in the ≤ 68 years group than in the ≥ 69 years group after gargling with mouthwash (P < 0.05). Similarly, after dividing the patients into two groups based on the median baseline HbA1c levels (≤ 7.4%, n = 92; ≥ 7.5%, n = 69), there was no significant difference in the change in the number of red complex species between the two groups after gargling with either water or mouthwash (Fig. 5B, Table 6). When examining sex differences (males, n = 105; females, n = 56), there was no significant difference in the change in the number of red complex species between the two groups after gargling with water. However, the number of red complex species was significantly lower in males than in females after gargling with mouthwash (P < 0.05) (Fig. 5C, Table 6). After dividing the patients into two groups based on the median disease duration (≤ 13 years, n = 87; ≥ 14 years, n = 74), no significant difference was observed in the number of red complex species between the two groups after gargling with either water or mouthwash (Fig. 5D, Table 6). Finally, when patients were divided by baseline BMI into normal (< 25.0 kg/m^2^, n = 110) and overweight (≥ 25.0 kg/m^2^, n = 51) groups, no significant difference was observed in the number of red complex species between the two groups after gargling with either water or mouthwash (Fig. 5E, Table 6).

**Table 6:** Changes in the number of red complex species as a function of clinical factors.

	Water gargling	Mouthwash gargling
Age (years)		
≤ 68	− 0.01 ± 0.08	− 0.66 ± 0.12
≥ 69	− 0.16 ± 0.09	− 0.32 ± 0.11*
HbA1c levels (%)		
≤ 7.4	− 0.12 ± 0.08	− 0.58 ± 0.12
≥ 7.5	− 0.04 ± 0.09	− 0.37 ± 0.11
Sex		
Male	− 0.08 ± 0.08	− 0.63 ± 0.11
Female	− 0.11 ± 0.10	− 0.24 ± 0.13^†^
Duration of T2DM (years)		
≤ 13	− 0.06 ± 0.08	− 0.47 ± 0.11
≥ 14	− 0.14 ± 0.09	− 0.51 ± 0.13
BMI (kg/m^2^)		
Normal	− 0.09 ± 0.08	− 0.52 ± 0.11
Overweight	− 0.11 ± 0.10	− 0.41 ± 0.13

Values are mean ± standard error of the mean.

*P < 0.05 compared with age ≤ 68 years.

^†^*P* < 0.05 compared with male sex in the mouthwash gargling group.

### Changes in the HbA1c levels as a function of clinical factors

**Figure 6:**
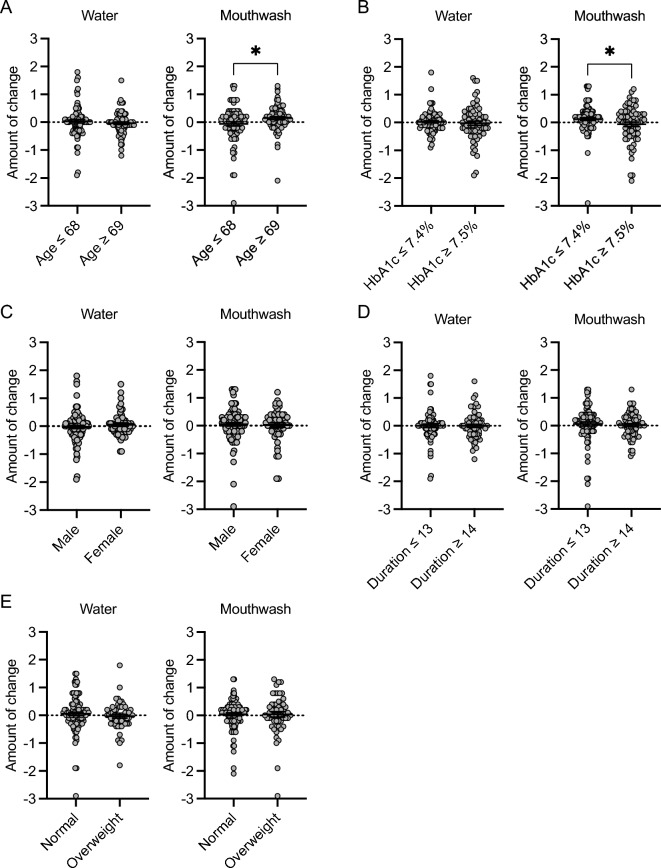
Comparison of changes in the HbA1c levels as a function of clinical factors. (**A**) Age, (**B**) baseline HbA1c level, (**C**) sex, (**D**) disease duration, and (**E**) baseline BMI. Significant differences were observed using Student’s t-test (*P < 0.05).

Changes in HbA1c levels were analyzed using a similar approach to that used to analyze changes in the number of red complex species in relation to each clinical factor. When classified by age, there was no significant difference in the change in the number of red complex species between the two groups after gargling with water. However, HbA1c levels were significantly lower in the ≤ 68 years group than in the ≥ 69 years group after gargling with mouthwash (P < 0.05) (Fig. 6A, Table 7). Similarly, when classified by baseline HbA1c levels, there was no significant difference in the change in the number of red complex species between the two groups after gargling with water. However, the HbA1c levels were significantly lower in the ≥ 7.5% group than in the ≤ 7.4% group after gargling with mouthwash (P < 0.05) (Fig. 6B, Table 7). There was no significant difference in the change in the number of red complex species in relation to sex, disease duration, or BMI after gargling with either water or mouthwash (Fig. 6C–E, Table 7).

**Table 7:** Changes in HbA1c levels as a function of clinical factors.

	Water gargling	Mouthwash gargling
Age (years)		
≤ 68	0.03 ± 0.06	− 0.06 ± 0.07
≥ 69	− 0.04 ± 0.05	0.15 ± 0.05*
HbA1c levels (%)		
≤ 7.4	0.02 ± 0.04	0.12 ± 0.05
≥ 7.5	− 0.02 ± 0.08	− 0.05 ± 0.08^†^
Sex		
Male	− 0.03 ± 0.05	0.05 ± 0.06
Female	0.06 ± 0.06	0.04 ± 0.08
Duration of T2DM (years)		
≤ 13	0.00 ± 0.06	0.06 ± 0.07
≥ 14	0.00 ± 0.05	0.02 ± 0.05
BMI (kg/m^2^)		
Normal	0.05 ± 0.06	0.04 ± 0.05
Overweight	− 0.03 ± 0.07	0.04 ± 0.10

Values are mean ± standard error of the mean (%).

*P < 0.05 compared with age ≤ 68 years.

^†^*P* < 0.05 compared with HbA1c levels ≤ 7.4% in the mouthwash gargling group.

## Discussion

T2DM is a metabolic disease characterized by chronic hyperglycemia caused by various degrees of inadequate β-cell insulin secretion and/or insulin resistance^29^. Patients with T2DM are at risk of developing complications because of long-term exposure to chronic hyperglycemia^30–32^. T2DM and periodontitis are now considered to be closely related because poor glycemic control leads to both an increased risk of alveolar bone loss and more severe progression of periodontitis^33^. Periodontitis and DM are mechanistically linked through an increase in pro-inflammatory mediators such as interleukin (IL)-1b, tumor necrosis factor, IL-6, and oxidative stress^34^. Because the incidence of periodontal disease is higher in patients with T2DM than in healthy individuals^35^, we decided to investigate the effect of mouthwash on major periodontopathic bacterial species and glycemic control in patients with T2DM.

Mouthwash is a self-care product that can be used easily without invasion of periodontal tissues^8^. Mouthwash containing chlorhexidine gluconate has antimicrobial effects on *P. gingivalis*^9^. Exposure to chlorhexidine gluconate induces detachment of red complex bacteria from the biofilm and decreased levels of living bacteria^36^. Mouthwash with 0.12% chlorhexidine gluconate is the recommended gargle after surgical procedures^37^. However, the gargle used in this study was developed with a lower concentration of chlorhexidine gluconate (0.00056%) and intended for daily use.

More than 90% of the patients in our study were able to gargle twice or three times per day, and gargling with mouthwash at these frequencies significantly reduced the number of red complex species in their oral cavity. Gargling with mouthwash containing chlorhexidine gluconate twice daily for 2 weeks has been reported to reduce dental plaque and gingival bleeding compared with gargling with water^38^. This finding, together with the findings of our study, suggest that gargling with mouthwash containing chlorhexidine gluconate at least twice per day can help inhibit periodontal disease and causative bacteria. Notably, the number of red complex species was not inhibited by gargling with water for as long as 6 months, indicating that gargling with water alone does not reduce red complex species, and that the antimicrobial effects of chlorhexidine gluconate are required to inhibit red complex species.

Professional periodontal treatments such as scaling, root planing, and antimicrobial therapy can reduce the number of red complex species and improve the glycemic control of patients with T2DM^22–24^. In addition, professional care with oral hygiene instructions results in good glycemic control of T2DM^25^. However, few reports have investigated the effects of self-care alone, including gargling, on the number of red complex species and HbA1c levels in patients with T2DM. We therefore evaluated the effects of gargling on these elements.

First, the changes in the number of red complex species and HbA1c levels after gargling with water or mouthwash were analyzed. The number of red complex species was not significantly reduced by gargling with water, but it was significantly reduced by gargling with mouthwash. However, HbA1c levels did not change significantly after gargling with either water or mouthwash. Although the case demonstrated in Fig. 2 was prominent and some cases did not so respond, we expect to be able to provide easily effective oral care for patients with diabetes if we can determine the ideal precondition.

In addition to seasonal variation as discussed earlier, several clinical factors such as age, sex, BMI, and disease duration influence the feasibility of treatment and the subsequent control of T2DM^39–43^. We therefore analyzed the effects of five clinical factors: age, baseline HbA1c levels, sex, disease duration, and baseline BMI on the relationship between gargling and the change in HbA1c levels^44–46^.

We initially analyzed the changes in the number of red complex species and HbA1c levels by age when gargling with water and mouthwash. Interestingly, the number of red complex species and HbA1c levels were significantly lower in younger patients (≤ 68 years) than in older patients (≥ 69 years) after gargling with mouthwash. It has been reported that older individuals accumulate more biofilm than younger individuals^44–46^, and older patients with T2DM might have difficulty controlling their blood glucose level because of increased systemic inflammation with aging^39,40^. These facts suggest that improvement of the oral environment through gargling with mouthwash could be attained in younger patients with T2DM.

We conducted similar analyses based on baseline HbA1c levels, which revealed no significant changes in the number of red complex species between the higher (≥ 7.5%) group and the lower (≤ 7.4%) group after gargling with either water or mouthwash. However, HbA1c levels were significantly lower in the higher group than in the lower group after gargling with mouthwash. In this study, the physicians were blinded to the PCR results until the completion of sample collection, and were free to adjust therapeutic agents as needed to improve glycemic control throughout the study. This strategy might have influenced the decrease in HbA1c levels in the higher group.

When we examined sex differences, only males showed a significant decrease in the number of red complex species while gargling with mouthwash. However, HbA1c values did not change markedly between males and females while gargling with either water or mouthwash. *P. gingivalis* is more abundant in the subgingival periodontal pockets of females than males^47^. Therefore, the volume and/or distribution of periodontopathic bacteria might attenuate the power of gargling with mouthwash from inhibiting red complex species in female patients.

This study has certain limitations. First, we only detected periodontopathic bacteria present in the oral cavity by PCR methods, and accurate counts or semi-quantification of bacteria were not performed. If semi-quantification or measurement of red complex bacteria could be performed, analyzing the correlation with changes in HbA1c levels would be possible. Second, this study was performed in an internal medicine clinic, and oral data such as periodontal pocket depth could not be collected. Third, we were unable to collect saliva at the same time in each patient and to strictly standardize the oral environment before saliva sampling, such as eating, drinking, or cleaning the mouth. The study period coincided with the middle of the COVID-19 pandemic and all patients wore masks. Checking the oral cavity of patients with T2DM who were highly susceptible to infection was difficult, even for a short time in a clinic visited by patients with many types of diseases. Finally, individual patients exhibited various patterns in the change of red complex species and glycemic control while gargling with mouthwash. One patient had a marked reduction in red complex species and a clear improvement in HbA1c levels. Another patient showed no changes in red complex species or HbA1c levels. These inconsistencies could be related to individual differences in factors, such as other systemic disease, medications, and the dental or periodontal status. Therefore, further studies should be planned, taking into account various patient factors to determine the effect of mouthwash gargling on the amount of red complex species and HbA1c levels in patients with T2DM.

In summary, T2DM patients can decrease red complex species by gargling with mouthwash two or three times a day, leading to possible improvement in glycemic control, especially in younger patients.

## Materials and methods

### Ethical approval

This study was conducted in full adherence to the Declaration of Helsinki. The study protocol was approved by the Ethics Committee of Osaka University Graduate School of Dentistry (Approval Number: R2-E16-2, approval date: November 6, 2020). Prior to sample collection, all subjects were informed of the study protocol, and provided written informed consent. This trial was registered with the University Hospital Medical Information Network Center (UMIN000049454). The study is reported in line with the STROBE statement (Supplementary table S1).

### Recruitment and selection of patients

The inclusion criteria for the study were patients with T2DM who regularly visited Itoh Internal Medicine Clinic (Toyonaka, Osaka, Japan), those who were not suffering from dementia, and those who were younger than 85 years old. The exclusion criteria were (1) < 6 species of periodontal pathogens, (2) HbA1c levels < 6.5%, and (3) a BMI ≥ 30.0 kg/m^2^. A diagnosis of T2DM was made on the basis of the criteria described in “Diagnosis and classification of diabetes mellitus”^48^. The patients were recruited from November 2020 to January 2021. Therefore, 350 patients with T2DM (245 males, 105 females; age: 34–85 years; mean age: 65.1 years) were initially enrolled in the study (Fig. 1). Saliva was collected from the 350 patients. A total of 224 patients (158 males, 66 females; age: 34–84 years; mean age: 65.8 years) who did not meet the exclusion criteria were selected for the study. Patients who did not give consent to continue the study or dropped out during the study were excluded. Finally, 193 subjects (133 males, 60 females; age: 34–84 years; mean age: 65.8 years) completed the study from January 2021 to June 2022. Of these, 20 patients with missing data were excluded. Data from the remaining 173 patients (115 males, 58 females; age: 34–84 years; mean age: 66.5 years) were analyzed.

### Study design

One hundred seventy-three patients were instructed to gargle with water three times a day for 6 months, followed by gargling three times a day with mouthwash for the next 6 months. During this experimental period, saliva specimens and blood samples were collected 6–12 times, every 1–2 months at each visit to the clinic. Bacterial DNA was extracted from the saliva samples to detect the three red complex species. Additionally, HbA1c levels were determined from blood samples.

### Methods of gargling with tap water or mouthwash

For the first 6 months, the patients were instructed to gargle with 25 mL water for 30 s three times a day (morning, afternoon, and night). For the next 6 months, they were instructed to gargle with mouthwash in the same manner. The mouthwash used in this study was ConCool F^®^ (Weltec Corp., Osaka, Japan), which contains 0.05% chlorhexidine gluconate. When gargling with the mouthwash, the patients were asked to dilute it to approximately 0.00056% (10 drops in 25 mL tap water for each gargle).

### Sample collection

Saliva specimens and blood samples were obtained 6–12 times at routine visits to the clinic every 1 or 2 months. HbA1c levels were determined using HPLC (ADAMS A1c HA-8182^®^, Arkray Inc., Kyoto, Japan) immediately after blood samples were collected at the clinic. Clinical factors including age, sex, disease duration, and BMI were collected from patients’ medical records.

### Detection of periodontopathic bacterial species

The distribution of 10 periodontopathic bacterial species was determined using previously developed PCR-based methods^49,50^. The primers used in this study are shown in Table 8^51–55^. Bacterial DNA was extracted from each oral specimen using Gentra Puregene Yeast/Bact. Kit B (Qiagen, Hilden, Germany). PCR analysis was then performed using a universal primer set targeting 16S rRNA genes to confirm that the bacterial DNA was successfully extracted. Subsequently, periodontopathic bacterial species were determined using respective specific primer sets. Amplification reactions were performed with 1 µl of template solution and Ex Taq DNA Polymerase (Takara Bio Inc., Otsu, Japan) in a total volume of 20 µl with the following cycling parameters, as described previously^50^: initial denaturation at 95 °C for 4 min; 30 cycles of 95 °C for 30 s, 60 °C for 30 s, and 72 °C for 30 s; and final extension at 72 °C for 7 min. Amplification reactions were performed in an iCycler thermal cycler (Bio-Rad Laboratories Inc., Hercules, CA, USA). The resulting products were separated by electrophoresis on a 1.5% agarose gel-Tris–acetate-EDTA buffer. The gels were stained with 0.5 μg/ml ethidium bromide and photographed under ultraviolet illumination using FAS-V (Nippon Genetics Co, Ltd., Tokyo, Japan).

**Table 8:** Primers used in this study.

Purpose	Sequence (5’-3’)	Size (bp)	References
Universal primer (positive control)			
PA	AGA GTT TGA TCC TGG CTC AG	315	^50^
PD	GTA TTA CCG CGG CTG CTG		
Detection of periodontitis-related species			
*Porphyromonas gingivalis*	CCG CAT ACA CTT GTA TTA TTG CAT GAT A	267	^50^
	AAG AAG TTT ACA ATC CTT AGG ACT GTC T		
*Treponema denticola*	AAG GCG GTA GAG CCG CTC A	311	^51^
	AGC CGC TGT CGA AAA GCC CA		
*Tannerella forsythia*	GCG TAT GTA ACC TGC CCG CA	641	^52^
	TGC TTC AGT GTC AGT TAT ACC T		
*Capnocytophaga ochracea*	AGA GTT TGA TCC TGG CTC AG	185	^53^
	GAT GCC GTC CCT ATA TAC TAT GGG G		
*Capnocytophaga sputigena*	AGA GTT TGA TCC TGG CTC AG	185	^53^
	GAT GCC GCT CCT ATA TAC CAT TAG G		
*Prevotella intermedia*	TTT GTT GGG GAG TAA AGC GGG	575	^53^
	TCA ACA TCT CTG TAT CCT GCG T		
*Prevotella nigrescens*	ATG AAA CAA AGG TTT TCC GGT AAG	804	^52^
	CCC ACG TCT CTG TGG GCT GCG A		
*Campylobact er rectus*	TTT CGG AGC GTA AAC TCC TTT TC	598	^52^
	TTT CTG CAA GCA GAC ACT CTT		
*Aggregatibacter actinomycetemcomitans*	CTA GGT ATT GCG AAA CAA TTT G	262	^54^
	CCT GAA ATT AAG CTG GTA ATC		
*Eikenella corrodens*	CTA ATA CCG CAT ACG TCC TAA G	688	^52^
	CTA CTA AGC AAT CAA GTT GCC C		

### Statistical analysis

The sample size was calculated using the G* Power program, version 3.1.9.7^56^. A value of 0.5 (Cohen’s d) was used for the sample size calculation to achieve > 80% power with a significance level of 5%. As a result, 51 or more patients were required for comparison between the subdivided groups. Statistical analyses were performed by using GraphPad Prism 9 (GraphPad Software Inc., La Jolla, CA, USA). Comparisons between the two groups were performed by using the chi-square test or Student’s t-test. Intergroup differences were estimated using analysis of variance with Bonferroni correction. Differences were considered statistically significant at *P* < 0.05.

### Supplementary Information

Supplementary Table S1.

## Data Availability

The data are available from the corresponding author upon reasonable request.
